# Bidirectional association between frailty and quality of life within English longitudinal study of aging

**DOI:** 10.1007/s11136-024-03809-7

**Published:** 2024-10-14

**Authors:** Ali Alattas, Farag Shuweihdi, Kate Best, Silviya Nikolova, Robert West

**Affiliations:** 1 Leeds Institute of Health Sciences, Faculty of Medicine and Health, University of Leeds, Leeds, UK; 2 Basic Science Department, College of Science and Health Professions, King Saud bin Abdulaziz for Health Sciences, Jeddah, Saudi Arabia

**Keywords:** Frailty, Quality of life, Bidirectional relationship, Latent curve model with structural residuals (LCM-SR)

## Abstract

**Purpose:**

The relationship between quality of life (QoL) and frailty has previously been investigated cross-sectionally and longitudinally as unidirectional where QoL depends upon frailty and where frailty depends on QoL. Here a bidirectional relationship is examined.

**Methods:**

This work uses a latent curve model with structured residuals to address the bidirectional association between QoL and frailty in older English people considering within-person and group levels. The study measures frailty using a functional frailty measure and quality of life using CASP-12. The sample size is 17,529.

**Results:**

There is a strong relationship between QoL (Quality of Life) and frailty, which is almost linear and inversely proportional over time. Although the cross-lagged coefficients from QoL to frailty and vice versa showed statistical significance, the impact was found to be minimal. The time between assessments (which are two years apart) and/or the few observations available per individual may have impacted the effect of this relationship. When accounting for gender, age, net wealth, and multimorbidity, some variations in the results were observed at the group level but not at the within-person level.

**Conclusion:**

The study provides empirical evidence that supports a bidirectional association between QoL and frailty in older individuals who reside at home. These results offer valuable insights for healthcare providers, as participants did not exhibit an advanced need for health services. Additionally, involving participants in evaluating and assessing these services enhances their effectiveness and overall benefit.

**Supplementary Information:**

The online version contains supplementary material available at 10.1007/s11136-024-03809-7.

## Abstract (plain language summary)

There’s growing evidence suggesting a two-way relationship between frailty and quality of life, meaning they can each affect the other. Understanding this dynamic requires sophisticated statistical methods, like the ones used in this study. Our research aims to shed light on a crucial question: when caring for older individuals, should our healthcare system prioritize improving quality of life or addressing frailty? Pinpointing which factor has a greater influence can guide healthcare institutions in allocating resources effectively and supporting elderly individuals in leading fulfilling lives as they age. Surprisingly, our findings reveal a minimal, yet significant, impact of frailty and quality of life on each other over time. This remained consistent across various demographic factors such as gender, age groups, socioeconomic status, and health conditions. One possible explanation for this minimal impact is the lengthy time gap between our measurements, spanning two years for each participant. These insights help us better understand how to care for our aging population by involving them in sharing their needs and thoughts. This input informs healthcare policies and practices, allowing us to better meet the diverse needs of older individuals who are raised at home.

**Supplementary Information:**

The online version contains supplementary material available at 10.1007/s11136-024-03809-7.

## Introduction

As a country’s population ages, as it does in many countries, there is a growing demand for healthcare services among older people [1]. Healthcare professionals work to identify factors that help older people maintain their independence and well-being [2]. This approach aligns with older people’s desires to live a satisfying life, maintain their quality of life (QoL), and avoid becoming frail [3].

Frailty is a clinical condition where the efficiency of the body systems and organs of the elderly decline, resulting in a higher likelihood of adverse outcomes when they are affected by minor diseases [4]. Frailty in older people increases the risk of several negative health outcomes, including disability, falls, hospitalization, institutionalization, and mortality [4]. Frailty progresses more rapidly with age, and higher frailty is also associated with reduced quality of life (QoL) [5].

The World Health Organization defined the QoL as “assesses individuals’ perception of their position in life in the context of culture, value systems, and their goals, expectations, standards, and concerns.” [6]. In this study, we will utilize the CASP-12 measure, which specifically focuses on non-health-related aspects such as control, autonomy, self-realization, and pleasure [7]. While health remains vital, we will evaluate it separately using a frailty index that encompasses both physical and mental health domains. This approach enables us to thoroughly investigate the connection between frailty and overall Quality of Life (QoL) in older adults residing in the community.

QoL is a multidimensional concept that includes psychological well-being, positive feelings, and functioning [8]. Gale, Cooper [9] discussed several studies that show that psychological well-being has an inverse relationship with ageing problems such as disability and survival. Improving quality of life was associated with increased life expectancy for older people worldwide [10]. Modern geriatric medicine aims to maintain a good quality of life by implementing appropriate interventions, as longevity does not necessarily ensure a high QoL in later life [11].

While previous studies reported a correlation between QoL and frailty, there are still some unanswered questions. Firstly, most of these studies have been cross-sectional in design, which leads to uncertainty regarding the direction of the effect [12]. Secondly, while some studies have explored the relationship between QoL and frailty using prospective cohorts, they have been unidirectional and inconsistent in their findings [12]. Some studies have reported frailty as a predictor of QoL [13, 14], while others have reported QoL as a predictor of frailty [9]. One study examined the bidirectional relationship between frailty and quality of life (QoL) for European participants using a cross-lagged panel model (CLPM) [15]. The study found that frailty and QoL have an inverse relationship, with frailty having a greater impact on this relationship. The study recommends early management of frailty to reduce the impact of low QoL on individuals and to provide an intervention plan. The main issue is that the study used a particular method that doesn’t look at differences between people and changes within individuals over time. This could mean the findings might not provide the full picture [16, 17]. To avoid any misleading conclusions, a latent curve model with structural residuals (LCM-SR) can be used. The LCM-SR approach was previously used to examine the bidirectional relationship between frailty and depression [18]. Insights from the findings from this type of analysis can inform healthcare providers and policymakers about interventions to manage and prevent frailty in older people living in the community.

This study aims to explore the relationship between quality of life (QoL) and frailty among older individuals in England. It investigates three hypotheses at the within-person level:

1. When frailty increases for a person at one time point, their quality of life tends to decrease at a later time point.
2. When the quality of life increases for a person at one time point, their frailty tends to decrease at a later time point.
3. At a specific point in time, a person’s increase in frailty is associated with a decrease in their quality of life.

At the group level, the study examines two hypotheses:

1. Participants who exhibit greater levels of frailty generally experience a reduced quality of life.
2. Participants who show increased trajectories in frailty tend to show decreased trajectories in QoL.

## Methods

### Study population

The English Longitudinal Study of Ageing (ELSA) is a comprehensive study that collects data from individuals aged over 50 years who reside in private households in England [19]. ELSA aims to accurately represent the ageing population in England by gathering information on three main aspects: health, social participation and wellbeing, and finances [20]. Over the past 18 years [21], ELSA has conducted ten waves of data collection, with new participants introduced in waves 3, 4, 6, 7, 9, and 10 to address sample attrition. Data are gathered using a combination of self-completion forms and face-to-face interviews [20]. This study utilized baseline and follow-up samples up to wave 9. Detailed information on the data collection schedule and sample sizes for each wave can be found in Table 1 of the supplementary information.

### Measures

#### Quality of life

Control, Autonomy, Self-realization, and Pleasure scale (CASP-12) was used to measure QoL. CASP-12 comprises four first-order factors (domains): Control, Autonomy, Self-realization and Pleasure. Three items for each domain and each item is scored 0 “never”, 1 “sometimes”, 2 “most often”, and 3 “often” [7]. Table 2 in the supplementary information shows the 12 items of the CASP-12. Item scores are added up to create total scale scores. The score of CASP-12 can range from 0 to 36, with higher scores indicating a better quality of life. The scores of the CASP-12 were divided by 36 (the highest score of the CASP-12) to be comparable with frailty measure scores.

#### Frailty

Functional frailty measure (FFM) was used to operationalize frailty [22]. It includes 44 self-reported deficits related to physical and mental health aspects. Table 4 in the supplementary information shows the 44 deficits of the FFM. Each item was coded as 0 if a deficit is not present or one if it is present. If a deficit has more than two values, we rescaled it between 0 and 1, for example the self-reported hearing items consist of a scale of five responses ranging from one to five, where one represents the worst and five represents the best. To standardize the responses, we converted each of the five options to a numerical value: one became 0, two became 0.25, three became 0.5, four became 0.75, and five became 1. The frailty score was computed by summing up the scores for each participant and dividing by the total number of valid of responses (at least 39 deficits were available). The frailty index ranges between 0 and 1, where higher scores indicate a higher frailty level.

#### Covariates

Four covariates were selected: gender, age, net wealth and long-term conditions (LTCs), which are defined as a condition which cannot be cured but can be managed through the use of medication and other therapies [23]. The participants were categorized into three net wealth levels (rich, average and poor), following the approach of Alattas, Nikolova [22]. In this work, the name for wealth levels is replaced with (high, medium and low). LTCs were categorized into two parts: non-multimorbid (zero or one LTC) and multimorbid (two or more LTCs). Based on Alattas, Nikolova [22] study, 16 health conditions were included: hypertension, angina, heart attack, congestive heart failure, abnormal heart rhythm, diabetes, stroke, lung disease, asthma, arthritis, osteoporosis, cancer, Parkinson’s, psychiatrist, Alzheimer’s, and dementia. Once the participants reported a LTC, the following observation were updated [22].

### Statistical analyses

#### Handling missing data in CASP-12

Dealing with missing data is crucial in longitudinal studies as it can cause biased and inefficient statistical analyses [24]. To handle missing data in CASP-12, we used two methods. Firstly, we filled in the missing values using information from within the missing group. For example, if an individual had a missing value between two reported waves for a deficit, the missing value for a deficit was replaced with the same value. Secondly, for any remaining missing values, we applied the MissForest algorithm [25]. Although it is a single imputation method, it accommodates the nonlinearities and interactions for predictors and is comparable to multiple imputation methods [26].

#### Modelling strategy

**Fig. 1:**
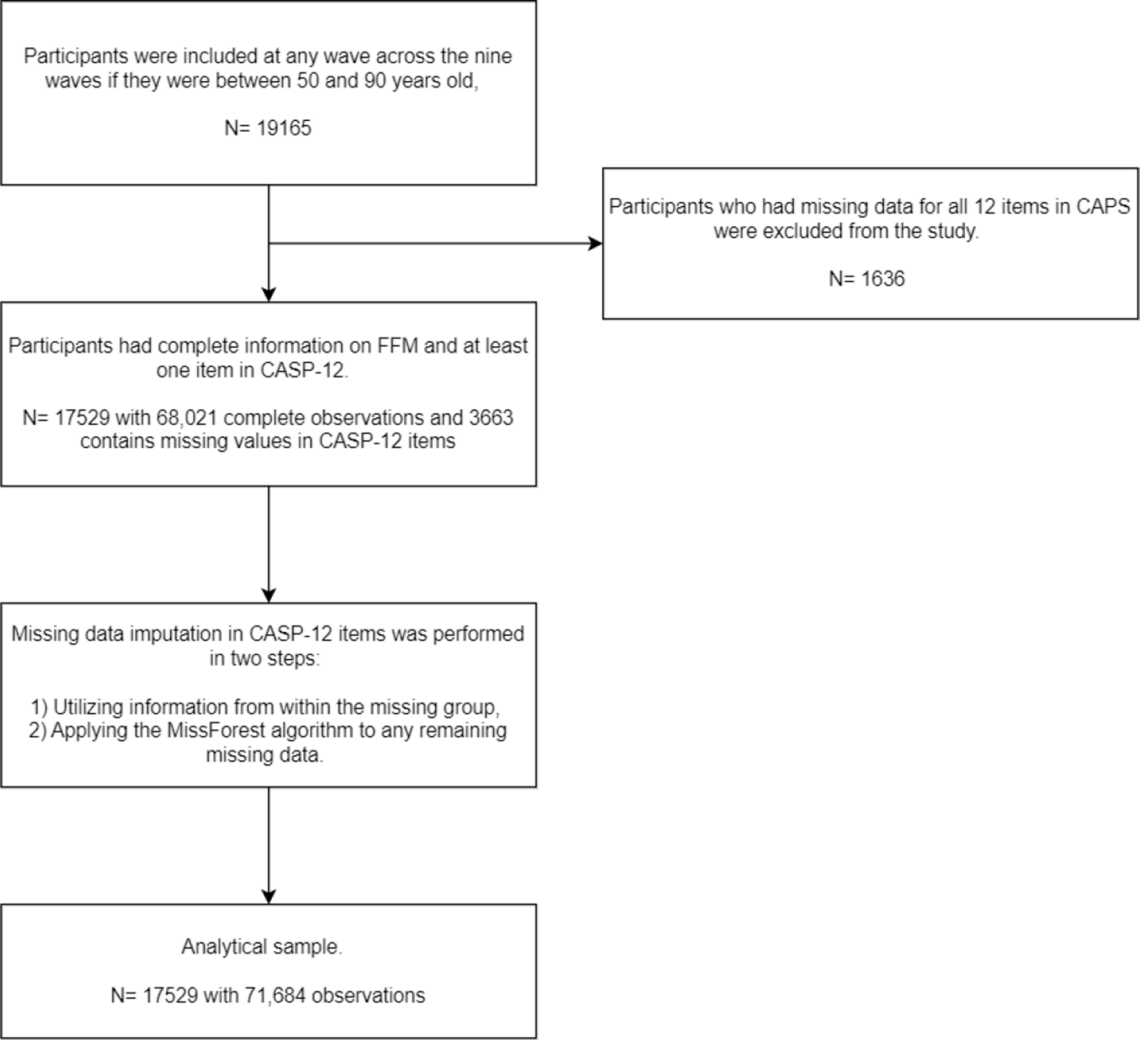
The procedures for the analytical sample

To test our hypotheses above, we used a latent curve model with structured residuals (LCM-SR) [16, 17]. LCM-SR is a modification of the autoregressive latent curve model to explicitly separate within-person and group levels effects by including a time-specific residual structure. Figure 1 shows an illustration plot for the relationship between the CASP-12 and FFM using the bivariate multivariate LCM-SR and three consecutive waves of the ELSA data. The growth curve component of this model captures group level variability in both the participant initial levels and trends, represented by the random intercept (RI) and the random slope (RS), respectively. The factor loadings on the RS factors are fixed to 0, 1 and 2 to reflect the weight of time of measurement and specify a positive linear trend for both constructs.

In this study, we have nine consecutive time points. The factor loadings for RS are fixed to 0, 1, 2, 3, 4, 5, 6, 7 and 8. The cross-lagged panel model component provides information on autoregressive, cross-lagged and within time association of the residuals. As an additional analysis, we conducted multiple group analyses on the final best-fit model in four variables: gender, two age groups (50–69 and 70–90), net wealth (high, medium and low), and multimorbidity (non-multimorbid and multimorbid).

A robust maximum likelihood estimator was used since the CASP-12 and FFM scores have skewness in some of the time points. Unbalanced samples across the nine waves handled with full information maximum likelihood (FIML) estimation. It estimates the model by using all information that is available from each participant, and it is a preferable approach under structural equation modelling [27]. For model development, two models of LCM-SR were tested. Firstly, only the random intercept (RI) factors are added for the scores of the CASP-12 and FFM, and the autoregression and cross-lagged parameters were constrained across the time points (Model A). Next, we added the random slope factors to the previous model as shown in Model B.

Three fit indices were considered: robust chi-square distribution with a degree of freedom (df), robust comparative fit index (RCFI) and robust root mean square error of approximation (RRMSEA). The Chi-square test is impacted by sample size, meaning that as the sample size grows, the test becomes more responsive to even minor variations between the correlation matrix of observed values and the correlation matrix of expected values. Alternatively, RCFI or RRMSEA were used to assess the goodness of fit. The RCFI and RRMSEA range from 0 to 1, and the values of 0.90 (acceptable fit) or 0.95 (good fit) are used as cut-points for the CFI while 0.06 (good fit) or 0.08 (acceptable fit) for RMSEA [28]. A sensitivity analysis was conducted on individuals with at least one complete set of both FFM and CASP-12 data across the nine waves. The sample size was 17,115. The analysis was performed in R software (4.3.3) and all of the CFA models (Models A and B) were estimated using the lavaan version 0.6–12 package [29].

## Results

### Sample construction

**Fig. 2:**
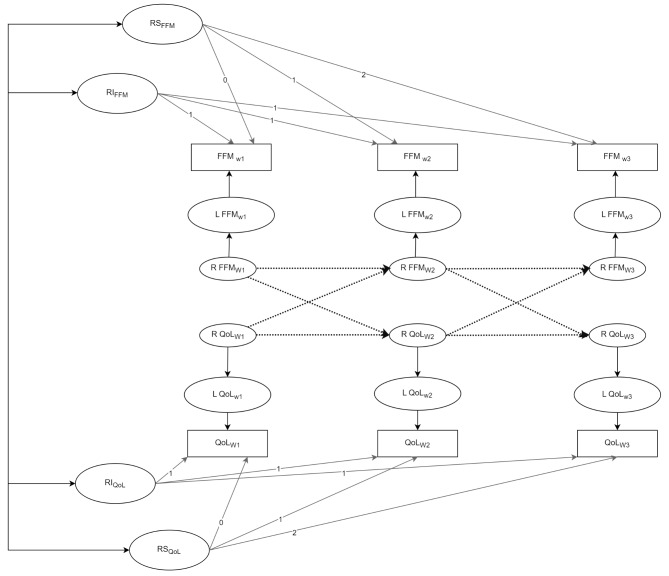
An illustration of the Latent curve model with Structured Residuals (LCM-SR) with random intercepts (RI) and slopes (RS) for three waves of MCASP-12 and FFM. The correlation within time points was deleted

In this study, 19,165 participants aged 50–90 reported at least one measure of frailty or QoL across the nine waves. The number of missing values for the frailty measure was quite small, while for the QoL measure, it was around 10 to 15% (see Table 3 in the supplementary information). Most of the missing values were for the whole CASP-12 items while others were for a part of the QoL items. As a result, the observations with missing values in the frailty measure were removed and the missing values for the whole CASP-12 items. Regarding the missing items on the QoL measure (1 to 11 items), two different methods were used sequentially to impute the missing values, see below, for the cases where one item at least responded to. The analytical sample size was 17,529. Figure 2 shows The procedures for the analytical sample.

Table 1 shows the sample size at each ELSA wave, ranging between 10,232 and 7034. There were 4128 (23.55%) present in one wave; two waves 2231 (12.73%); three waves: 1955 (11.15%); four waves: 1623 (9.26%); five waves: 1392 (7.94%); six waves: 1861 (10.62%); seven waves: 1220 (6.96%); eight waves: 1168 (6.66%); nine waves: 1951 (11.13%). Table 1 shows that most participants were female, and the average age at wave 1 was 64, and 68 at wave 9. Nearly, half were wealthy across the nine waves. The prevalence of multimorbidity increased over time. The average score of the CASP-12 was 26, and the average scores of FFM was 0.16. Women, older individuals, lower net wealth, more chronic conditions, frailty and low QoL were the characteristics of the missing data in CASP-12 (See Table 5 in the supplementary information). Pairwise correlations and reliability estimates for the FMM and CASP-12 scores across the nine waves are shown in Table 6 in the supplementary information.

**Table 1:** Summary statistics for the sample across the nine waves

	Wave	1	2	3	4	5	6	7	8	9
*N* = 17,529		10,232	7972	7774	8924	8725	8691	7920	7034	7244
Age (mean (SD))		64.51 (9.87)	65.55 (9.39)	64.89 (9.93)	65.14 (9.16)	66.47 (8.91)	66.54 (9.11)	67.29 (9.15)	68.75 (8.77)	68.07 (9.72)
Gender n(%)	Female	5591 (54.6)	4438 (55.7)	4291 (55.2)	4919 (55.1)	4853 (55.6)	4814 (55.4)	4406 (55.6)	3934 (55.9)	4047 (55.9)
	Male	4641 (45.4)	3534 (44.3)	3483 (44.8)	4005 (44.9)	3872 (44.4)	3877 (44.6)	3514 (44.4)	3100 (44.1)	3197 (44.1)
Net Wealth n(%)	High	4147 (40.5)	3300 (41.4)	3227 (41.5)	3712 (41.6)	3539 (40.6)	3565 (41.0)	3242 (40.9)	2897 (41.2)	2973 (41.0)
	Medium	2078 (20.3)	1655 (20.8)	1560 (20.1)	1779 (19.9)	1748 (20.0)	1782 (20.5)	1585 (20.0)	1437 (20.4)	1478 (20.4)
	Low	4007 (39.2)	3017 (37.8)	2987 (38.4)	3433 (38.5)	3438 (39.4)	3344 (38.5)	3093 (39.1)	2700 (38.4)	2793 (38.6)
Long-term conditions	0	2898 (28.3)	1846 (23.2)	1823 (23.4)	2164 (24.2)	1818 (20.8)	1816 (20.9)	1599 (20.2)	1219 (17.3)	1427 (19.7)
	1	3302 (32.3)	2432 (30.5)	2295 (29.5)	2636 (29.5)	2427 (27.8)	2368 (27.2)	2046 (25.8)	1760 (25.0)	1772 (24.5)
	2+	4029 (39.4)	3685 (46.2)	3653 (47.0)	4115 (46.1)	4476 (51.3)	4505 (51.8)	4273 (54.0)	4049 (57.6)	4043 (55.8)
	NA	3 (0.0)	9 (0.1)	3 (0.0)	9 (0.1)	4 (0.0)	2 (0.0)	2 (0.0)	6 (0.1)	2 (0.0)
FFM (mean (SD))		0.16 (0.14)	0.16 (0.13)	0.15 (0.13)	0.15 (0.13)	0.16 (0.13)	0.15 (0.13)	0.15 (0.13)	0.15 (0.13)	0.15 (0.12)
CASP-12 (mean (SD))		26.85 (5.94)	27.00 (6.06)	25.90 (5.88)	25.89 (5.88)	25.93 (5.99)	25.77 (6.04)	26.48 (5.97)	26.47 (6.02)	26.64 (6.02)

**Fig. 3:**
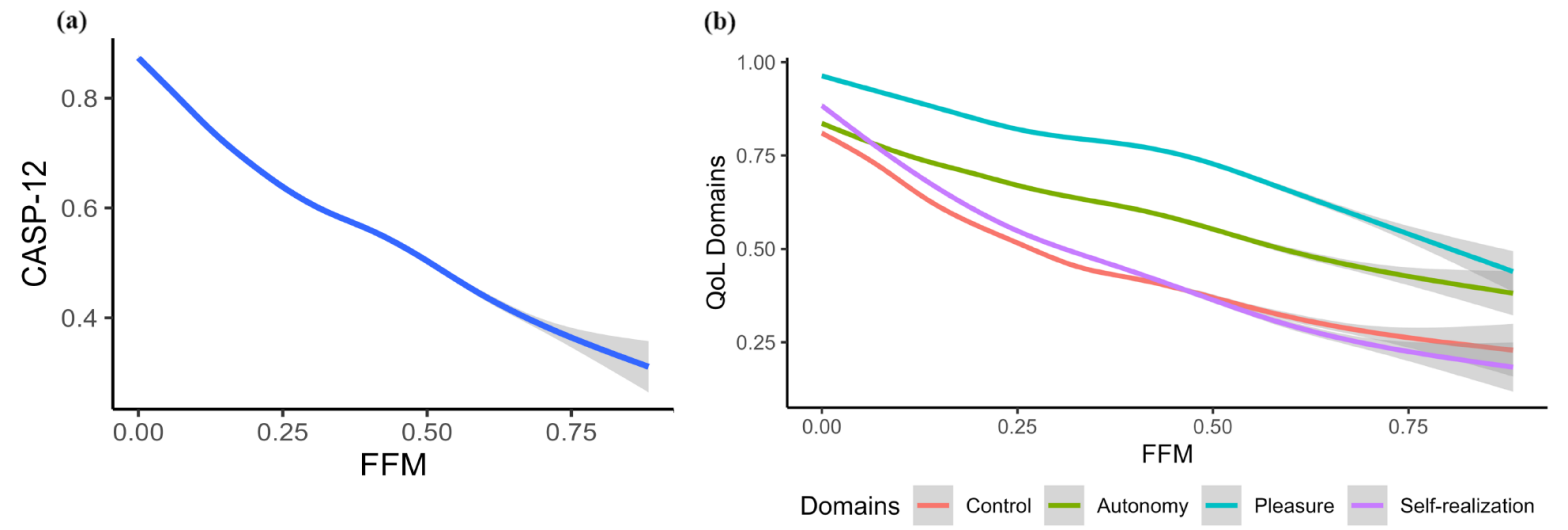
An inverse correlation between CASP-12 and functional frailty measure (FFM) (**a**) and an inverse correlation between the four domains of CASP-12 and FFM (**b**)

Figure 3 displays an inverse correlation between CASP-12 and its four domains with FFM. Notably, the control and self-realization domains have a more pronounced decline. Table 2 shows the model fit indices for Model A and B. Notably adding RS factors, as shown in Model B, shows an improved fit. We did not find any improvement in the model fits when autoregression estimation and cross-lagged parameters varied across the nine waves (not shown here).

**Table 2:** Fit statistics of two LCM-SR models

Model	Chi-s	df	RTLI	RCFI	RRMSEA
A	3163.361*	160	0.968	0.967	0.067 (0.065–0.069)
B	1574.042*	151	0.985	0.986	0.045 (0.043–0.048)

Model A: random intercept (RI) factors are added for both the CASP-12 and FFM; Model B random slope factors were added for CASP-12 and FFM to the model A. *p-value < 0.01

Table 3 shows the parameter estimations of Models A and B. At the within-person level (autoregressive, cross-lagged and correlation within-person level), Model A shows higher effects than Model B. Regarding the cross-lagged effect in Model B, the results indicated that the relationship between prior frailty and later QoL was stronger than between early QoL and subsequent frailty. An increase of one unit of the standard deviation of the CASP-12 predicts a negative change to FFM at a later time or vice versa by around 4%. Moreover, there is a moderate inverse correlation between CASP-12 and FFM within the same time points of the participants in the two models (Models A and B). In other words, the participants who tend to score a high level of QoL with one unit of standard deviation also tend to have a low score with one unit of frailty and vice versa.

**Table 3:** Standardized parameters for models a and B

Model	A	B
Random effect: Means		
CASP-12 intercept** ×36	0.718* 25.85	0.739* 26.60
FFM intercept**	0.165*	0.150*
CASP-12 slope** ×36	--	-0.006* -0.22
FFM slope**	--	0.005*
**Random effect: Correlation**		
CASP-12 intercept vs. FFM Intercept	-0.700*	-0.707*
CASP-12 intercept vs. CASP-12 slope	--	-0.110*
CASP-12 intercept vs. FFM slope	--	0.140*
FFM intercept vs. CASP-12 slope	--	-0.020
FFM intercept & FFM slope	--	0.101*
CASP-12 slope & FFM slope	--	-0.795*
**Autoregressive CASP-12 to CASP-12**		
Wave 2	0.318*	0.201*
Wave 3	0.329*	0.222*
Wave 4	0.312*	0.211*
Wave 5	0.303*	0.207*
Wave 6	0.290*	0.197*
Wave 7	0.300*	0.206*
Wave 8	0.292*	0.204*
Wave 9	0.302*	0.211*
**Autoregressive FFM to FFM**		
Wave 2	0.404*	0.224*
Wave 3	0.399*	0.223*
Wave 4	0.397*	0.227*
Wave 5	0.381*	0.216*
Wave 6	0.380*	0.219*
Wave 7	0.384*	0.222*
Wave 8	0.370*	0.213*
Wave 9	0.369*	0.209*
**Cross-lagged CASP-12 to FFM**		
Wave 2	-0.123*	-0.036*
Wave 3	-0.121*	-0.038*
Wave 4	-0.115*	-0.036*
Wave 5	-0.111*	-0.035*
Wave 6	-0.109*	-0.035*
Wave 7	-0.114*	-0.037*
Wave 8	-0.108*	-0.036*
Wave 9	-0.110*	-0.034*
**Cross-lagged FFM to CASP-12**		
Wave 2	-0.145*	-0.041*
Wave 3	-0.151*	-0.043*
Wave 4	-0.149*	-0.044*
Wave 5	-0.145*	-0.042*
Wave 6	-0.140*	-0.041*
Wave 7	-0.141*	-0.041*
Wave 8	-0.138*	-0.040*
Wave 9	-0.144*	-0.042*
**Association within-wave**		
Wave 1	-0.475*	-0.273*
Wave 2	-0.317*	-0.203*
Wave 3	-0.314*	-0.203*
Wave 4	-0.311*	-0.203*
Wave 5	-0.307*	-0.202*
Wave 6	-0.304*	-0.202*
Wave 7	-0.306*	-0.203*
Wave 8	-0.303*	-0.202*
Wave 9	-0.303*	-0.202*

** unstandardized; *p < 0.001

Regarding group level effects, Model B provided further information on the effects of the random factors. First, the initial levels of CASP-12 and FFM are around 27 (0.739 × 36 = 26.60) and 0.15, respectively. On average, there is a linear decrease over time in the CASP-12 score by 0.22 (0.006 × 36) per wave, as well as a linear increase over time by 0.005 in the FFM score between two consecutive waves. Also, the correlation of RI factors between CASP-12 and FFM, as well as the correlation of RS factors between CASP-12 and FFM, show a *stronger* negative relationship. The sensitivity analysis results were similar to those of the main analysis (see Tables 7 and 8 in the supplementary information).

### Multiple group analysis

The model group’s analysis was based on model B’s specifications. The models that included gender showed differences in the within-person effects. The autoregressive parameters of CASP-12 across the nine waves were higher for females, while the autoregressive parameters of FFM were higher for males (see Table 8 in the supplementary information). Thus, previous CASP-12 scores will have a greater impact on later CASP-12 scores in females, while FFM scores will have a higher impact on later FFM scores in males. Additionally, the cross-lagged parameters from CASP-12 to FFM were higher for males, while the cross-lagged parameters from FFM to CASP-12 were higher for females. Thus, previous CASP-12 scores will have a greater impact on later FFM scores in males, while FFM scores will have a higher impact on later CASP-12 scores in females. At the group level, the means of the RI for CASP-12 were similar in both genders, around 27. The mean of RI for FFM was higher in females than in males, with 0.15 and 0.13, respectively. The means of RS factors for CASP-12 and FFM were similar in both genders. The inverse correlation between RS factors for CASP-12 and FFM was higher in males (see Table 10 in the supplementary information).

Regarding multiple group analysis for age, the autoregressive parameters of FFM across the nine waves were higher for the oldest participants (70–90). Additionally, the cross-lagged parameters from CASP-12 to FFM and vice versa were higher for the most senior participants across the nine waves. The inverse association between CASP-12 and FFM within-person level at the same time point is similar for the participant age (50–69) and the group of oldest participants age (70–90) across the nine waves. At the group level, the means of the RI for CASP-12 were similar in both age groups. However, the mean of RI for FFM was higher in oldest than older participants, with 0.15 and 0.13, respectively. Also, the means of the RS factors differed for CASP-12 and FFM: both RSs tend to be steeper in the oldest participants. The inverse correlation between the RI and the RS factors for CASP-12 and FFM was higher in the oldest participants (see Table 10 in the supplementary information). So, age impacts the inverse relationship between frailty and QoL, and it is more pronounced among the oldest participants.

Regarding multiple group analysis for net wealth, the autoregressive parameters of CASP-12 and FFM across the nine waves were higher for participants with low net wealth. Additionally, the cross-lagged parameters from CASP-12 to FFM and vice versa were higher for participants with medium net wealth. The inverse association between CASP-12 and FFM within-person level simultaneously is higher for medium and low net wealth participants across the nine waves. At the group level, participants with low net wealth had the lowest mean RI for CASP-12, while the highest mean RI was for FFM. The participants with low net wealth tended to have steeper RS means for CASP-12 and larger FFM means. The inverse correlation of RI between CASP-12 and FFM and RS between CASP-12 and FFM were higher for participants with low net wealth (see Table 10 in the supplementary information).

Multiple group analysis concerning LTCs (zero/one vs. 2+) showed that the autoregressive parameters of FFM across the nine waves were higher for multimorbid participants. Additionally, the cross-lagged parameters from CASP-12 to FFM were higher for multimorbid, while the cross-lagged parameters from FFM to CASP-12 were higher for non-multimorbid participants. The inverse association between CASP-12 and FFM within-person level at the same point is higher for multimorbid participants across the nine waves. At the group level, the mean of RI for CASP-12 was higher for non-multimorbid participants, and the means of RS for CASP-12 decreased less for non-multimorbid participants, while the means of RI and RS for FFM were higher for multimorbid participants. The inverse correlation of RI between CASP-12 and FFM and RS between CASP-12 and FFM were higher for multimorbid participants (see Table 11 in the supplementary information). The observations with missing data for LTCs were omitted.

## Discussion

This study explored how quality of life (QoL) and frailty are related among 17,529 English individuals over a 16-year period. The relationship between QoL, measured by CASP-12, and frailty, assessed by FFM, showed a consistent inverse pattern, almost linear. Despite statistically significant cross-lagged coefficients between CASP-12 and FFM, indicating mutual influence over time, the practical longitudinal impact was seen as minimal.

Some of the results presented in Table 1 are consistent with previous studies that have utilized ELSA data. For example, Marshall, Nazroo [30] reported summary statistics for a sample of ELSA participants at wave 1, which showed an average age of 65, 54% of females, and a frailty score of 0.16. Similarly, Niederstrasser, Rogers [31] reported summary statistics for a sample of ELSA participants at wave 2, which showed an average age of 67, a distribution of wealth categories similar to our findings, and a frailty score of 0.16 at wave 2. Differences in cohort samples and frailty measures may have contributed to these slight differences. The analytical samples of these studies do not match our analytical sample, which means that comparison of results with theirs might not be appropriate. Most sample studies did not include refreshment samples in later waves. Marshall, Nazroo [30], excluded younger participants (i.e., < 60) [5] or presented the summary statistics for the sample by classified them by a variable group, such as gender [32] or survival [5]. In our study, long-term conditions (LTCs) were defined based on 16 health conditions, which differs from other studies that use ELSA. For instance, Nguyen, Chua [32] included 26 health conditions in their study at wave 2 in ELSA and reported that around 80% of participants had two or more LTCs.

There are few studies that investigate the two-way relationship between frailty and quality of life (QoL) in observational studies [15]. A cross-lagged panel model (CLPM) is one method that can be used to examine this kind of investigation [16, 17] although biased estimations might occur when an individual’s characteristics are not distinguished from those of the sample group. Our findings suggest that there is a minimal but significant bidirectional relationship between frailty and QoL, indicating that neither has a dominant effect on the other. The study revealed that the connection between frailty and QoL, as influenced by social background, is relevant under current conditions. This challenges the idea that one factor causes changes in the other and suggests that both contribute to the level of successful ageing at any given time. The consistent negative correlation between frailty and QoL over nine different time points supports this conclusion. Consequently, social care professionals may inquire with individuals in need of assistance to determine the specific support they require. It appears that women typically sought support to maintain or improve their QoL, while men tended to seek support to prevent severe frailty.

Additionally, it was observed that factors such as gender, age, net wealth, and multimorbidity had a significant impact on the relationship between Quality of Life (QoL) and frailty at a group level but were not as noticeable at the individual level. This indicates that although these factors play a role in the average relationship between QoL and frailty, they vary more when considering individual experiences over time. This could be due to limited available information, such as the few observations per individual. The long intervals (two years apart) between measurements and sample attrition caused by unobserved reasons like hospitalization or death could be other reasons. As far as we know, this is the first study to adjust the bidirectional association between frailty and QoL based on gender, age, net wealth, and LTCs.

A recent study showed that CASP-12, a shorter version of CASP-19, more independently captures quality of life [7]. It is critical to consider age ranges − 50–59, 60–69, and 70+ - to avoid measurement errors when using CASP-12. So, applying multi-group analysis is recommended if the range age of participants is beyond one of these three age groups [33].

The results of this study have important clinical and research implications. Clinically, the findings suggest that interventions aimed at addressing frailty and quality of life (QoL) may need to be more adaptable and dynamic, tailored to different stages of aging. Early interventions like physical rehabilitation or social support could potentially help prevent temporary declines in QoL due to frailty, and vice versa. However, once a certain level of frailty is reached, the relationship between frailty and QoL may stabilize or diminish, requiring a focus on broader health determinants [34, 35]. From a research perspective, longitudinal studies are essential to understanding how the relationship between frailty and QoL evolves over time, aiding in the prediction of outcomes and the development of long-term care strategies [15]. Additionally, these findings can inform public health policies, emphasizing the need for community-based programs focused on maintaining or improving QoL among older adults, particularly those at risk of or experiencing frailty.

The present study has notable strengths. It is the first study to investigate the reciprocal relationship between quality of life (QoL) and frailty over 16 years with a considerable sample size using the LCM-SR method. The quality of the dataset was excellent. The analysis was adjusted for several crucial factors, including sex, age, wealth, and long-term conditions (LTCs).

One of the limitations of this study is the presence of missing data in CASP-12 items. We addressed this issue by utilizing two imputation methods sequentially and we employed FIML to handle unbalanced samples across the nine waves under the structural equation model framework. Also, observations that were dropped out did not have any CASP-12 items, accounting for around 9% of the target sample. These individuals were more frail, older, and had less wealth. The impact of the bias was reduced by applying multiple group analysis. The time between measurements was two years, so more immediate impacts are not captured. The sample had a higher proportion of wealthier individuals although our analyses demonstrated that the results remain robust against net wealth differences. Although the sample size was large, we cannot assume generalizability in this work since participant weighting was not adjusted for representativeness. Another limitation is the reliance on self-reported measures of frailty without a physical test. While self-reported frailty and QoL can introduce bias due to inaccurate reporting or recall issues [36, 37], for instance, in studies relying solely on self-reported data, there is a risk of measurement error and bias that can affect the validity and reliability of the findings. On the other hand, it also serves as a strength by offering direct insights into the participants’ experiences, which is particularly relevant for studies focusing on quality of life and subjective health measures.

In addition, one factor that could influence the bidirectional relationship between frailty and quality of life is loneliness, especially among older adults in Great Britain. Research highlights the importance of considering diverse patterns of loneliness when developing policies and interventions to combat social isolation and improve the quality of life for older adults living in the community [38].

To summarize, a bidirectional relationship between QoL and frailty is close to linear and inversely proportional over time. Although the bidirectional cross-lagged for CASP-12 and FFM coefficients were statistically significant, the magnitude of the effects are small. Even when we considered factors like gender, age, wealth, and having multiple health conditions, we noticed some differences in the overall results between different people, but less within the same person over time. The study provides empirical evidence supporting a bidirectional association between QoL and frailty in older individuals who reside at home, providing valuable insights for healthcare providers to modify their services accordingly.

## Electronic supplementary material

Below is the link to the electronic supplementary material.

Supplementary Material 1

Supplementary Material 2

## Data Availability

Researchers can access the ELSA dataset for free from the UK Data Service. You can assess the data here https://beta.ukdataservice.ac.uk/datacatalogue/series/series?id=200011.
