# Supplementary material for: Bidirectional association between frailty and quality of life within English longitudinal study of aging
Source: Qual Life Res. 2024 Oct 14;34(1):261–71. doi: 10.1007/s11136-024-03809-7 (PMC11802669; doi:10.1007/s11136-024-03809-7)
Supplement: Supplementary file 2 — Supplementary Material 2 [file 11136_2024_3809_MOESM2_ESM.docx]

**Bidirectional Association Between Frailty and Quality of Life within English Longitudinal Study of Aging**

**Applied research in QoL Journal**

Ali Alattas^1,2^*, Farag Shuweihdi^1^, Kate Best^1^, Silviya Nikolova^1^, Robert West^1^

1 Leeds Institute of Health Sciences, Faculty of Medicine and Health, University of Leeds, Leeds, UK.

2 Basic Science Department, College of Science and Health Professions, King Saud bin Abdulaziz for Health Sciences, Jeddah, Saudi Araiba.

*Corresponding author

Email: [mmaalat@leeds.ac.uk](mailto:mmaalat@leeds.ac.uk)

Table 1: Collected data years of ELSA and their sample size

|  | Wave1 | Wave2 | Wave3 | Wave4 | Wave 5 | Wave 6 | Waveb7 | Wave 8 | Wave 9 |
| --- | --- | --- | --- | --- | --- | --- | --- | --- | --- |
| Collected data year | 2002/3 | 2004/5 | 2006/7 | 2008/9 | 2010/11 | 2012/13 | 2014/15 | 2016/17 | 2018/19 |
| N | 12099 | 9432 | 9771 | 11050 | 10274 | 10601 | 9666 | 8445 | 8736 |

Table 2: Summary statistics for the 19165 participants aged 50-90, including all those who participated in at least one wave of FFM or CASP-12 in the ELSA data set across the nine waves.

|  | Wave | 1 | 2 | 3 | 4 | 5 | 6 | 7 | 8 | 9 |
| --- | --- | --- | --- | --- | --- | --- | --- | --- | --- | --- |
| N= 19165 |  | 11426 | 9062 | 9227 | 10612 | 9970 | 10215 | 9334 | 8223 | 8429 |
| Age (mean (SD)) |  | 64.85 (10.05) | 66.00 (9.68) | 65.02 (10.27) | 65.37 (9.46) | 66.77 (9.22) | 66.60 (9.43) | 67.33 (9.40) | 68.77 (8.97) | 67.96 (9.99) |
| Gender  n(%) | Female | 6224 (54.5) | 5015 (55.3) | 5056 (54.8) | 5796 (54.6) | 5488 (55.0) | 5578 (54.6) | 5136 (55.0) | 4543 (55.2) | 4661 (55.3) |
|  | Male | 5202 (45.5) | 4047 (44.7) | 4171 (45.2) | 4816 (45.4) | 4482 (45.0) | 4637 (45.4) | 4198 (45.0) | 3680 (44.8) | 3768 (44.7) |
| Net Wealth  n(%) | High | 4483 (39.2) | 3590 (39.6) | 3628 (39.3) | 4156 (39.2) | 3923 (39.3) | 4044 (39.6) | 3672 (39.3) | 3262 (39.7) | 3352 (39.8) |
|  | Average | 2253 (19.7) | 1804 (19.9) | 1818 (19.7) | 2075 (19.6) | 1967 (19.7) | 2007 (19.6) | 1833 (19.6) | 1629 (19.8) | 1670 (19.8) |
|  | Low | 4491 (39.3) | 3546 (39.1) | 3549 (38.5) | 4100 (38.6) | 3870 (38.8) | 3951 (38.7) | 3605 (38.6) | 3216 (39.1) | 3268 (38.8) |
|  | NA | 199 ( 1.7) | 122 ( 1.3) | 232 ( 2.5) | 281 ( 2.6) | 210 ( 2.1) | 213 ( 2.1) | 224 ( 2.4) | 116 ( 1.4) | 139 ( 1.6) |
| Long-term conditions  n(%) | 0 | 3186 (27.9) | 2054 (22.7) | 2144 (23.2) | 2559 (24.1) | 2054 (20.6) | 2139 (20.9) | 1878 (20.1) | 1414 (17.2) | 1679 (19.9) |
|  | 1 | 3663 (32.1) | 2711 (29.9) | 2687 (29.1) | 3061 (28.8) | 2729 (27.4) | 2731 (26.7) | 2361 (25.3) | 2006 (24.4) | 2035 (24.1) |
|  | 2+ | 4567 (40.0) | 4284 (47.3) | 4385 (47.5) | 4970 (46.8) | 5168 (51.8) | 5333 (52.2) | 5086 (54.5) | 4790 (58.3) | 4708 (55.9) |
|  | NA | 10 ( 0.1) | 13 ( 0.1) | 11 ( 0.1) | 22 ( 0.2) | 19 ( 0.2) | 12 ( 0.1) | 9 ( 0.1) | 13 ( 0.2) | 7 ( 0.1) |
| FFM | (mean (SD)) | 0.17 (0.15) | 0.18 (0.14) | 0.16 (0.15) | 0.16 (0.14) | 0.17 (0.15) | 0.16 (0.15) | 0.16 (0.15) | 0.16 (0.15) | 0.16 (0.14) |
|  | NA n(%) | 4 | 2 | 0 | 3 | 5 | 0 | 0 | 0 | 0 |
| CASP-12 ca | (mean (SD)) | 26.96 (5.88) | 27.22 (5.91) | 25.99 (5.84) | 25.96 (5.85) | 26.00 (5.96) | 25.88 (5.98) | 26.59 (5.94) | 26.59 (5.95) | 26.74 (5.96) |
|  | NA n(%) | 1790 (15.7) | 1769 (19.5) | 1744 (18.9) | 1965 (18.5) | 1483 (14.9) | 1959 (19.2) | 1799 (19.3) | 1498 (18.2) | 1525 (18.1) |
|  | 1 | 387 ( 3.4) | 335 ( 3.7) | 190 ( 2.1) | 178 ( 1.7) | 176 ( 1.8) | 291 ( 2.8) | 232 ( 2.5) | 183 ( 2.2) | 225 ( 2.7) |
|  | 2 | 80 ( 0.7) | 96 ( 1.1) | 52 ( 0.6) | 24 ( 0.2) | 37 ( 0.4) | 66 ( 0.6) | 57 ( 0.6) | 51 ( 0.6) | 45 ( 0.5) |
|  | 3 | 45 ( 0.4) | 54 ( 0.6) | 14 ( 0.2) | 15 ( 0.1) | 14 ( 0.1) | 18 ( 0.2) | 26 ( 0.3) | 27 ( 0.3) | 24 ( 0.3) |
|  | 4 | 41 ( 0.4) | 36 ( 0.4) | 8 ( 0.1) | 11 ( 0.1) | 10 ( 0.1) | 17 ( 0.2) | 19 ( 0.2) | 10 ( 0.1) | 10 ( 0.1) |
|  | 5 | 30 ( 0.3) | 41 ( 0.5) | 9 ( 0.1) | 9 ( 0.1) | 6 ( 0.1) | 12 ( 0.1) | 15 ( 0.2) | 16 ( 0.2) | 8 ( 0.1) |
|  | 6 | 19 ( 0.2) | 32 ( 0.4) | 7 ( 0.1) | 11 ( 0.1) | 6 ( 0.1) | 10 ( 0.1) | 12 ( 0.1) | 11 ( 0.1) | 15 ( 0.2) |
|  | 7 | 15 ( 0.1) | 28 ( 0.3) | 4 ( 0.0) | 7 ( 0.1) | 6 ( 0.1) | 12 ( 0.1) | 6 ( 0.1) | 12 ( 0.1) | 8 ( 0.1) |
|  | 8 | 26 ( 0.2) | 19 ( 0.2) | 10 ( 0.1) | 10 ( 0.1) | 11 ( 0.1) | 14 ( 0.1) | 11 ( 0.1) | 6 ( 0.1) | 3 ( 0.0) |
|  | 9 | 7 ( 0.1) | 27 ( 0.3) | 7 ( 0.1) | 7 ( 0.1) | 4 ( 0.0) | 3 ( 0.0) | 12 ( 0.1) | 8 ( 0.1) | 6 ( 0.1) |
|  | 10 | 13 ( 0.1) | 21 ( 0.2) | 6 ( 0.1) | 12 ( 0.1) | 5 ( 0.1) | 12 ( 0.1) | 13 ( 0.1) | 7 ( 0.1) | 7 ( 0.1) |
|  | 11 | 16 ( 0.1) | 24 ( 0.3) | 5 ( 0.1) | 6 ( 0.1) | 9 ( 0.1) | 18 ( 0.2) | 13 ( 0.1) | 7 ( 0.1) | 9 ( 0.1) |

Table 3: 12 items of CASP12 and its four domains

| Item (number) | **Domain** |
| --- | --- |
|  |  |
| My age prevents me from doing the things I would like to do (*C1*) | Control |
| I feel that what happens to me is beyond my control (*C2*) |  |
| I feel left out of things (*C3*) |  |
| I can do the things I want to do (*A1*) | Autonomy |
| I feel that I can please myself what I do (*A2*) |  |
| A lack of money stops me from doing things I want to do (*A3*) |  |
| I look forward to each day (*P1*) | Pleasure |
| I feel that my life has meaning (*P2*) |  |
| I enjoy the things that I do (*P3*) |  |
| I feel full of energy these days (*S1*) | Self-realisation |
| I feel that life is full of opportunities (*S2*) |  |
| I feel that the future looks good for me (*S3*) |  |

Table 4: 44 deficits of the functional frailty measure (FFM)

| Domain |  | Item | Domain |  | Item |
| --- | --- | --- | --- | --- | --- |
| Mobility | 1 | difficulty walking 100 yards | Psychology | 24 | Whether felt depressed much of the time during the past week |
|  | 2 | difficulty sitting 2 hours |  | 25 | Whether felt everything they did during the past week was an effort |
|  | 3 | difficulty getting up from a chair after sitting long periods |  | 26 | felt their sleep was restless during the past week |
|  | 4 | difficulty climbing several flights of stairs without resting |  | 27 | Whether was happy much of the time during the past week |
|  | 5 | difficulty climbing one flight stairs without resting |  | 28 | Whether felt lonely much of the time during the past week |
|  | 6 | difficulty stooping, kneeling or crouching |  | 29 | Whether enjoyed life much of the time during the past week |
|  | 7 | difficulty reaching or extending arms above shoulder level |  | 30 | Whether felt sad much of the time during the past week |
|  | 8 | difficulty pulling or pushing large objects |  | 31 | Whether could not get going much of the time during the past week |
|  | 9 | difficulty lifting or carrying weights over 10 pounds | self-reported and operation | 32 | Self-reported eyesight |
|  | 10 | difficulty picking up 5p coin from the table |  | 33 | Self-reported general health |
|  | 11 | difficulty dressing, including putting on shoes and socks |  | 34 | Self-reported hearing |
|  | 12 | difficulty walking across a room |  | 35 | Fallen down |
|  | 13 | difficulty bathing or showering |  | 36 | fractured hip |
|  | 14 | difficulty eating, such as cutting up food |  | 37 | had joint replacement |
|  | 15 | difficulty getting in and out of bed |  | 38 | had pain whilst walking |
|  | 16 | difficulty using the toilet, including getting up or down | memory test | 39 | correct day of month given |
|  | 17 | difficulty using a map to figure out how to get around a strange place |  | 40 | correct month given |
|  | 18 | preparing a hot meal |  | 41 | correct year given |
|  | 19 | shopping for groceries |  | 42 | correct day given |
|  | 20 | making telephone calls |  | 43 | Number of words recalled immediately |
|  | 21 | taking medications |  | 44 | Number of words recalled after a delay |
|  | 22 | doing work around the house or garden |  |  |  |
|  | 23 | managing money, such as bills and expenses |  |  |  |

Table 5: Summary statistics for participants with and without missing data in CASP-12.

| CASP-12 items |  | Complete observations | Missing observations |  |
| --- | --- | --- | --- | --- |
|  |  | 68021 | 3663 |  |
| Age (mean (SD)) |  | 66.17 (9.19) | 72.11 (9.55) | <0.001 |
| gender (%) | Female | 37428 ( 55.0) | 2373 ( 64.8) | <0.001 |
|  | Male | 30593 ( 45.0) | 1290 ( 35.2) |  |
| Net Wealth (%) | High | 28838 ( 42.4) | 914 ( 25.0) | <0.001 |
|  | Average | 13748 ( 20.2) | 780 ( 21.3) |  |
|  | Low | 24401 ( 35.9) | 1914 ( 52.3) |  |
|  | NA | 1034 ( 1.5) | 55 ( 1.5) |  |
| LTCs (mean (SD)) | 0 | 15095 ( 22.2) | 491 ( 13.4) | <0.001 |
|  | 1 | 19282 ( 28.3) | 868 ( 23.7) |  |
|  | 2 | 33608 ( 49.4) | 2297 ( 62.7) |  |
|  | NA | 36 ( 0.1) | 7 ( 0.2) |  |
| FI44 (mean (SD)) |  | 0.15 (0.13) | 0.22 (0.15) | <0.001 |
| CASP-12 |  | 26.45 (5.91) | 24.29 (6.69) | <0.001 |

Table 6: Pairwise correlations and reliability estimates for the FMM and CASP-12 scores across the nine waves.

|  | **FFM_W1_** | **FFM_W2_** | **FFM_W3_** | **FFM_W4_** | **FFM_W5_** | **FFM_W6_** | **FFM_W7_** | **FFM_W8_** | **FFM_W9_** | **CASP_W1_** | **CASP_W2_** | **CASP_W3_** | **CASP_W4_** | **CASP_W5_** | **CASP_W6_** | **CASP_W7_** | **CASP_W8_** | **CASP_W9_** |
| --- | --- | --- | --- | --- | --- | --- | --- | --- | --- | --- | --- | --- | --- | --- | --- | --- | --- | --- |
| **FFM_W1_** | (0.91) | 0.794 | 0.755 | 0.707 | 0.686 | 0.664 | 0.630 | 0.608 | 0.565 | -0.596 | -0.537 | -0.495 | -0.472 | -0.474 | -0.456 | -0.408 | -0.410 | -0.376 |
| **FFM_W2_** | -- | (0.91) | 0.813 | 0.760 | 0.730 | 0.694 | 0.654 | 0.637 | 0.574 | -0.538 | -0.597 | -0.533 | -0.507 | -0.497 | -0.473 | -0.430 | -0.426 | -0.391 |
| **FFM_W3_** | -- | -- | (0.91) | 0.796 | 0.750 | 0.726 | 0.672 | 0.645 | 0.586 | -0.512 | -0.535 | -0.568 | -0.514 | -0.510 | -0.477 | -0.422 | -0.446 | -0.423 |
| **FFM_W4_** | -- | -- | -- | (0.90) | 0.815 | 0.773 | 0.734 | 0.705 | 0.651 | -0.450 | -0.482 | -0.506 | -0.577 | -0.542 | -0.503 | -0.467 | -0.463 | -0.440 |
| **FFM_W5_** | -- | -- | -- | -- | (0.91) | 0.818 | 0.766 | 0.740 | 0.689 | -0.450 | -0.478 | -0.481 | -0.524 | -0.600 | -0.546 | -0.505 | -0.500 | -0.489 |
| **FFM_W6_** | -- | -- | -- | -- | -- | (0.91) | 0.824 | 0.783 | 0.726 | -0.407 | -0.424 | -0.454 | -0.489 | -0.529 | -0.579 | -0.521 | -0.517 | -0.504 |
| **FFM_W7_** | -- | -- | -- | -- | -- | -- | (0.91) | 0.820 | 0.756 | -0.395 | -0.405 | -0.408 | -0.460 | -0.490 | -0.519 | -0.563 | -0.528 | -0.513 |
| **FFM_W8_** | -- | -- | -- | -- | -- | -- | -- | (0.90) | 0.801 | -0.357 | -0.385 | -0.397 | -0.440 | -0.476 | -0.494 | -0.514 | -0.583 | -0.554 |
| **FFM_W9_** | -- | -- | -- | -- | -- | -- | -- | -- | (0.91) | -0.328 | -0.361 | -0.364 | -0.389 | -0.433 | -0.449 | -0.469 | -0.520 | -0.583 |
| **CASP_W1_** | -- | -- | -- | -- | -- | -- | -- | -- | -- | (0.88) | 0.704 | 0.661 | 0.616 | 0.598 | 0.558 | 0.537 | 0.512 | 0.507 |
| **CASP_W2_** | -- | -- | -- | -- | -- | -- | -- | -- | -- | -- | (0.89) | 0.729 | 0.681 | 0.651 | 0.617 | 0.580 | 0.564 | 0.542 |
| **CASP_W3_** | -- | -- | -- | -- | -- | -- | -- | -- | -- | -- | -- | (0.89) | 0.764 | 0.722 | 0.682 | 0.651 | 0.623 | 0.591 |
| **CASP_W4_** | -- | -- | -- | -- | -- | -- | -- | -- | -- | -- | -- | -- | (0.89) | 0.765 | 0.718 | 0.683 | 0.652 | 0.613 |
| **CASP_W5_** | -- | -- | -- | -- | -- | -- | -- | -- | -- | -- | -- | -- | -- | (0.89) | 0.768 | 0.722 | 0.682 | 0.661 |
| **CASP_W6_** | -- | -- | -- | -- | -- | -- | -- | -- | -- | -- | -- | -- | -- | -- | (0.89) | 0.758 | 0.718 | 0.685 |
| **CASP_W7_** | -- | -- | -- | -- | -- | -- | -- | -- | -- | -- | -- | -- | -- | -- | -- | (0.89) | 0.761 | 0.724 |
| **CASP_W8_** | -- | -- | -- | -- | -- | -- | -- | -- | -- | -- | -- | -- | -- | -- | -- | -- | (0.89) | 0.768 |
| **CASP_W9_** | -- | -- | -- | -- | -- | -- | -- | -- | -- | -- | -- | -- | -- | -- | -- | -- | -- | (0.89) |

CASP here means CASP-12; Reliability estimates based on tetrachoric correlations are reported in the diagonal and marked with coefficient ω in parenthesis.

Table 7: Fit statistics of two LCM-SR models (Complete cases in FFM or CASP-12); N= 17115

| Model | Chi-s | df | RTLI | RCFI | RRMSEA |
| --- | --- | --- | --- | --- | --- |
| A | 3037.01* | 160 | 0.968 | 0.967 | 0.068 (0.065 - 0.070) |
| B | 1570.48* | 151 | 0.985 | 0.985 | 0.047 (0.044- 0.050) |

Model A: random intercept (RI) factors are added for both the CASP-12 and FFM; Model B random slope factors were added for CASP-12 and FFM to the model A. *p-value<0.01.

Table 8: Standardized parameters for Models A and B (Complete cases in FFM or CASP-12)

| Model | A | B |
| --- | --- | --- |
| **Random effect: Means** | | |
| CASP-12 intercept**  ×36 | 0.721*  25.96 | 0.741*  26.68 |
| FFM intercept** | 0.162* | 0.148* |
| CASP-12 slope**  ×36 | -- | -0.006*  -0.22 |
| FFM slope** | -- | 0.004* |
| **Random effect: Correlation** |  |  |
| CASP-12 intercept vs FFM Intercept | -0.699* | *-0.700** |
| CASP-12 intercept vs CASP-12 slope | -- | -0.118* |
| CASP-12 intercept vs FFM slope | -- | 0.130* |
| FFM intercept vs CASP-12 slope | -- | -0.032 |
| FFM intercept & FFM slope | -- | 0.090* |
| CASP-12 slope & FFM slope | -- | *-0.766** |
| **Autoregressive CASP-12 to CASP-12** |  |  |
| Wave 2 | 0.324* | 0.197* |
| Wave 3 | 0.323* | 0.207* |
| Wave 4 | 0.310* | 0.201* |
| Wave 5 | 0.303* | 0.199* |
| Wave 6 | 0.293* | 0.191* |
| Wave 7 | 0.301* | 0.198* |
| Wave 8 | 0.297* | 0.195* |
| Wave 9 | 0.303* | 0.203* |
| **Autoregressive FFM to FFM** |  |  |
| Wave 2 | 0.409* | 0.234* |
| Wave 3 | 0.393* | 0.222* |
| Wave 4 | 0.397* | 0.232* |
| Wave 5 | 0.379* | 0.219* |
| Wave 6 | 0.380* | 0.224* |
| Wave 7 | 0.383* | 0.226* |
| Wave 8 | 0.371* | 0.218* |
| Wave 9 | 0.366* | 0.212* |
| **Cross-lagged CASP-12 to FFM** |  |  |
| Wave 2 | -0.118* | -0.032* |
| Wave 3 | -0.113* | -0.032* |
| Wave 4 | -0.109* | -0.032* |
| Wave 5 | -0.105* | -0.031* |
| Wave 6 | -0.104* | -0.030* |
| Wave 7 | -0.107* | -0.032* |
| Wave 8 | -0.105* | -0.031* |
| Wave 9 | -0.102* | -0.030* |
| **Cross-lagged FFM to CASP-12** |  |  |
| Wave 2 | -0.146* | -0.045* |
| Wave 3 | -0.146* | -0.045* |
| Wave 4 | -0.146* | -0.046* |
| Wave 5 | -0.142* | -0.045* |
| Wave 6 | -0.139* | -0.044* |
| Wave 7 | -0.139* | -0.044* |
| Wave 8 | -0.136* | -0.043* |
| Wave 9 | -0.141* | -0.045* |
| **Association within-wave** |  |  |
| Wave 1 | -0.478* | -0.294* |
| Wave 2 | -0.330* | -0.215* |
| Wave 3 | -0.322* | -0.214* |
| Wave 4 | -0.321* | -0.215* |
| Wave 5 | -0.317* | -0.214* |
| Wave 6 | -0.315* | -0.214* |
| Wave 7 | -0.317* | -0.214* |
| Wave 8 | -0.314* | -0.214* |
| Wave 9 | -0.313* | -0.214* |

** unstandardized ; *p<0.001;

Table 9: Model fit statistics of B model for multi-group (age, net wealth and LTCs)

|  | N | Chi-s | df | RTLI | RCFI | R-RMSEA |
| --- | --- | --- | --- | --- | --- | --- |
| **Gender** |  |  |  |  |  |  |
| Male | 8007 | 695.024* | 151 | 0.986 | 0.986 | 0.044 (0.040-0.049) |
| Female | 9527 | 1042.820* | 151 | 0.985 | 0.985 | 0.047 (0.043-0.050) |
| **Age** |  |  |  |  |  |  |
| 50-69 | 13570 | 1043.626* | 151 | 0.984 | 0.984 | 0.047 (0.043-0.051) |
| 70-90 | 8337 | 634.338 | 151 | 0.985 | 0.985 | 0.044 (0.038-0.049) |
| **Net wealth** |  |  |  |  |  |  |
| High | 8409 | 818.510* | 151 | 0.982 | 0.982 | 0.048 (0.044-0.052) |
| Average | 6315 | 419.212* | 151 | 0.988 | 0.988 | 0.038 (0.026-0.050) |
| Low | 9559 | 586.923* | 151 | 0.988 | 0.988 | 0.039 (0.034-0.044) |
| **Multimorbidity** |  |  |  |  |  |  |
| Non-multimorbid | 11475 | 875.734 | 151 | 0.977 | 0.977 | 0.052 (0.047-0.057) |
| multimorbid | 9831 | 806.335 | 151 | 0.986 | 0.986 | 0.043 (0.039-0.047) |

(Model B) the random slope factors were added to the model A.

Table 10: Standardized parameters for Model B in three group variables; gender, two age groups and three net wealth groups

|  | **Gender** | | **Age** | | **Net wealth** | | |
| --- | --- | --- | --- | --- | --- | --- | --- |
| Parameter | Male | Female | 50-69 | 70-90 | High | Average | Low |
| **Random effect: Means** |  |  |  |  |  |  |  |
| CASP-12 intercept**  ×36 | 0.733*  26.39 | 0.743*  26.75 | 0.743*  26.75 | 0.739*  26.60 | 0.784*  28.22 | 0.749*  26.96 | 0.696*  25.10 |
| FFM intercept** | 0.139* | 0.160* | 0.139* | 0.170* | 0.112* | 0.141 | 0.187* |
| CASP-12 slope**  ×36 | -0.006*  -0.22 | -0.007*  -0.25 | -0.002*  -0.07 | -0.01*  -0.36 | -0.005*  -0.18 | -0.005*  -0.18 | -0.005*  -0.18 |
| FFM slope** | 0.004* | 0.005* | 0.001* | 0.007* | 0.004* | 0.003* | 0.004* |
| **Random effect: Correlation** |  |  |  |  |  |  |  |
| CASP-12 intercept vs FFM Intercept | -0.722* | -0.710* | -0.683* | -0.755* | -0.635* | -0.601* | -0.727* |
| CASP-12 intercept vs CASP-12 slope | -0.092* | -0.119* | -0.095^ | -0.118^ | -0.069 | -0.203* | -0.193* |
| CASP-12 intercept vs FFM slope | 0.189* | 0.108* | 0.137* | 0.150* | 0.143* | 0.199* | 0.237* |
| FFM intercept vs CASP-12 slope | 0.021 | -0.048 | -0.016 | 0.183* | -0.163* | -0.049 | 0.185* |
| FFM intercept & FFM slope | --0.188* | -0.040 | -0.217* | -0.248* | -0.078 | -0.245* | -0.247* |
| CASP12 slope & FFM slope | -0.818* | -0.783* | -0.630* | -0.846* | -0.703* | -0.644* | -0.888* |
| **Autoregressive CASP-12 to CASP-12** |  |  |  |  |  |  |  |
| Wave 2 | 0.192* | 0.208* | 0.208* | 0.201* | 0.184* | 0.174* | 0.226* |
| Wave 3 | 0.211* | 0.230* | 0.218* | 0.239* | 0.204* | 0.186* | 0.259* |
| Wave 4 | 0.197* | 0.222* | 0.223* | 0.206* | 0.198* | 0.185* | 0.231* |
| Wave 5 | 0.198* | 0.215* | 0.217* | 0.202* | 0.189* | 0.182* | 0.241* |
| Wave 6 | 0.184* | 0.207* | 0.201* | 0.204* | 0.186* | 0.181* | 0.215* |
| Wave 7 | 0.196* | 0.214* | 0.224* | 0.189* | 0.189* | 0.170* | 0.237* |
| Wave 8 | 0.191* | 0.213* | 0.208* | 0.207* | 0.188* | 0.195* | 0.226* |
| Wave 9 | 0.203* | 0.218* | 0.216* | 0.211* | 0.203* | 0.177* | 0.239* |
| **Autoregressive FFM to FFM** |  |  |  |  |  |  |  |
| Wave 2 | 0.261* | 0.202* | 0.194* | 0.286* | 0.180* | 0.203* | 0.249* |
| Wave 3 | 0.262* | 0.197* | 0.196* | 0.285* | 0.194* | 0.219* | 0.232* |
| Wave 4 | 0.259* | 0.207* | 0.207* | 0.271* | 0.200* | 0.222* | 0.236* |
| Wave 5 | 0.253* | 0.192* | 0.190* | 0.273* | 0.185* | 0.206* | 0.233* |
| Wave 6 | 0.246* | 0.200* | 0.192* | 0.275* | 0.199* | 0.207* | 0.228* |
| Wave 7 | 0.272* | 0.192* | 0.205* | 0.270* | 0.187* | 0.228* | 0.237* |
| Wave 8 | 0.231* | 0.200* | 0.183* | 0.280* | 0.186* | 0.203* | 0.218* |
| Wave 9 | 0.257* | 0.180* | 0.193* | 0.253* | 0.178* | 0.213* | 0.227* |
| **Cross-lagged CASP-12 to FFM** |  |  |  |  |  |  |  |
| Wave 2 | -0.031* | -0.039* | -0.020* | -0.072* | -0.025* | -0.065* | -0.035* |
| Wave 3 | -0.032* | -0.040* | -0.021* | -0.077* | -0.027* | -0.068* | -0.036* |
| Wave 4 | -0.030* | -0.040* | -0.022* | -0.065* | -0.026* | -0.071* | -0.033* |
| Wave 5 | -0.030* | -0.038* | -0.020* | -0.064* | -0.025* | -0.064* | -0.033* |
| Wave 6 | -0.028* | -0.039* | -0.020* | -0.065* | -0.026* | -0.066* | -0.032* |
| Wave 7 | -0.031* | -0.039* | -0.023* | -0.064* | -0.026* | -0.068* | -0.035* |
| Wave 8 | -0.028* | -0.040* | -0.020* | -0.071* | -0.026* | -0.069* | -0.032* |
| Wave 9 | -0.029* | -0.037* | -0.021* | -0.065* | -0.024* | -0.065* | -0.033* |
| **Cross-lagged FFM to CASP-12** |  |  |  |  |  |  |  |
| Wave 2 | -0.032* | -0.046* | -0.032* | -0.064* | -0.038* | -0.058* | -0.028* |
| Wave 3 | -0.034* | -0.048* | -0.033* | -0.072* | -0.043* | -0.062* | -0.029* |
| Wave 4 | -0.033* | -0.049* | -0.034* | -0.070* | -0.044* | -0.063* | -0.028* |
| Wave 5 | -0.033* | -0.047* | -0.032* | -0.070* | -0.042* | -0.060* | -0.029* |
| Wave 6 | -0.032* | -0.046* | -0.031* | -0.070* | -0.042* | -0.057* | -0.027* |
| Wave 7 | -0.033* | -0.045* | -0.033* | -0.064* | -0.040* | -0.061* | -0.028* |
| Wave 8 | -0.031* | -0.045* | -0.030* | -0.066* | -0.040* | -0.063* | -0.026* |
| Wave 9 | -0.036* | -0.045* | -0.032* | -0.067* | -0.043* | -0.064* | -0.029* |
| **Association within-wave** |  |  |  |  |  |  |  |
| Wave 1 | -0.261* | -0.283* | -0.311* | -0.281* | -0.241* | -0.304* | -0.306* |
| Wave 2 | -0.191* | -0.211* | -0.205* | -0.204* | -0.164* | -0.235* | -0.224* |
| Wave 3 | -0.192* | -0.212* | -0.205* | -0.206* | -0.166* | -0.236* | 0.225* |
| Wave 4 | -0.191* | -0.212* | -0.206* | -0.203* | -0.166* | -0.236* | 0.224* |
| Wave 5 | -0.191* | -0.211* | -0.205* | -0.203* | -0.170* | -0.235* | 0.224* |
| Wave 6 | -0.190* | -0.211* | -0.204* | -0.203* | -0.165* | -0.235* | 0.222* |
| Wave 7 | -0.192* | -0.211* | -0.206* | -0.202* | -0.165* | -0.236* | 0.224* |
| Wave 8 | -0.190* | -0.211* | -0.204* | -0.204* | -0.165* | -0.236* | 0.222* |
| Wave 9 | -0.191* | -0.210* | -0.205* | -0.202* | -0.165* | -0.235* | 0.224* |

** unstandardized ;*p<0.001; ^p<0.05

Table 11: Standardized parameters for Model B (two long-term conditions samples)

| Parameter | Non-multimorbid | multimorbid |
| --- | --- | --- |
| N |  |  |
| **Random effect: Means** |  |  |
| CASP-12 intercept**  ×36 | 0.770*  27.72 | 0.699*  25.16 |
| FFM intercept** | 0.114* | 0.200 |
| CASP-12 slope**  ×36 | -0.004*  -0.14 | -0.006*  -0.22 |
| FFM slope** | 0.001* | 0.004* |
| **Random effect: Correlation** |  |  |
| CASP-12 intercept vs FFM Intercept | -0.612* | -0.719* |
| CASP-12 intercept vs CASP-12 slope | -0.006 | -0.234* |
| CASP-12 intercept vs FFM slope | 0.251* | 0.264* |
| FFM intercept vs CASP-12 slope | -0.198* | 0.135* |
| FFM intercept & FFM slope | -0.269* | -0.301* |
| CASP-12 slope & FFM slope | -0.564* | -0.794* |
| **Autoregressive MCASP12 to MCASP12** |  |  |
| Wave 2 | 0.208* | 0.219* |
| Wave 3 | 0.231* | 0.238* |
| Wave 4 | 0.219* | 0.224* |
| Wave 5 | 0.223* | 0.216* |
| Wave 6 | 0.203* | 0.212* |
| Wave 7 | 0.218* | 0.214* |
| Wave 8 | 0.209* | 0.219* |
| Wave 9 | 0.230* | 0.217* |
| **Autoregressive FFM to FFM** |  |  |
| Wave 2 | 0.192* | 0.227* |
| Wave 3 | 0.181* | 0.213* |
| Wave 4 | 0.178* | 0.220* |
| Wave 5 | 0.179* | 0.208* |
| Wave 6 | 0.174* | 0.212* |
| Wave 7 | 0.186* | 0.214* |
| Wave 8 | 0.174* | 0.207* |
| Wave 9 | 0.178* | 0.201* |
| **Cross-lagged CASP-12 to FFM** |  |  |
| Wave 2 | -0.036* | -0.059* |
| Wave 3 | -0.039* | -0.059* |
| Wave 4 | -0.037* | -0.056* |
| Wave 5 | -0.037* | -0.053* |
| Wave 6 | -0.035* | -0.054* |
| Wave 7 | -0.039* | -0.055* |
| Wave 8 | -0.038* | -0.055* |
| Wave 9 | -0.040* | -0.052* |
| **Cross-lagged FFM to CASP-12** |  |  |
| Wave 2 | -0.054* | -0.040* |
| Wave 3 | -0.053* | -0.041* |
| Wave 4 | -0.053* | -0.042* |
| Wave 5 | -0.054* | -0.040* |
| Wave 6 | -0.050* | -0.040* |
| Wave 7 | -0.051* | -0.039* |
| Wave 8 | -0.047* | -0.039* |
| Wave 9 | -0.051* | -0.040* |
| **Association within-wave** |  |  |
| Wave 1 | -0.265* | -0.312* |
| Wave 2 | -0.173* | -0.225* |
| Wave 3 | 0.174* | -0.225* |
| Wave 4 | 0.173* | -0.225* |
| Wave 5 | 0.173* | -0.223* |
| Wave 6 | 0.172* | -0.223* |
| Wave 7 | 0.173* | -0.224* |
| Wave 8 | 0.173* | -0.224* |
| Wave 9 | 0.174* | -0.223* |

** unstandardized ;*p<0.01
